# The Application of Wireless Network-Based Artificial Intelligence Robots in Badminton Teaching and Training

**DOI:** 10.1155/2022/3910307

**Published:** 2022-06-02

**Authors:** Shouling Gao

**Affiliations:** School of Physical Education and Health, Changsha Medical University, Changsha 410219, Hunan, China

## Abstract

Artificial intelligence technology has already set its foot in various industries, including sports, to train athletes. In this research article, people will study the application of wireless networks based on artificial intelligence robots in badminton teaching and training. People propose a system that deploys intelligent robots to teach badminton to athletes. The robots will train the players with various moves and techniques required for the game. The wireless networking system allows the robot to connect to the network. Various sets of plays and players' movements were preprogrammed for the robot. The trainer has to select essential factors such as training mode and set height required for a particular player in the robot—these are the complexities in badminton training. Moreover, in the case of effective and efficient training, people need a robot that will aid in different training modes. The changing variables, such as speed, frequency, angle, height, and change in coordinates, are utilised in the training and teaching of robots, which are more efficient than the traditional training methods given by people. The decision tree algorithm (DTA) is used in this research and is compared with the existing sports motion segmentation method (SMSM). From the results, it is observed that the proposed DTA has given improved accuracy of 93% compared with the SMSM.

## 1. Introduction

It was not until the 1920s that badminton was brought to China, and as the years went by, it became a popular sport. It is not uncommon for pupils to have to learn badminton in physical education classes, although the science behind the sport is rudimentary at best. Pupils with no prior badminton experience confront two challenges: first, they have to deal with more difficult badminton abilities; second, they have to deal with somewhat tedious explanations; while describing difficult methods, students are quickly side-tracked and lack enthusiasm for learning [1].

When teaching badminton in college and university classrooms, people are more than likely dealing with college students. It is impossible to build a strong badminton education from the ground up if the basis of badminton technology is so thin or nonexistent. Second, pupils are easily side-tracked and have a lack of enthusiasm for studying when they are presented with technical explanations that are tedious and difficult to understand [2]. Finally, collegiate badminton technical instruction is required. In the face of such a huge class, it is hard for the instructor to consider each student's level of acceptance within the short period of time they have to teach. When it comes to the number of badminton teaching hours available at different colleges and universities, most only provide it for one semester. Badminton is a sport that requires a significant amount of time to learn and master. It is difficult for pupils with a low level of receptivity to keep pace with educational advancement. Because of this, an intelligent badminton teaching system based on a neural network is very important.

To teach badminton in an effective manner, specialists both domestically and internationally conduct extensive studies [3]. There is a lot of intelligent teaching research going on outside of the United States and Europe, including Japan, Canada, and other nations like those mentioned above. Stanford, MIT, Memphis, Carnegie Mellon, and California are among the American colleges that have built prototype intelligent systems. As an example, the US National Science Foundation has allocated $22.50 million to the study of intelligent systems that aid in learning and creativity in humans and other living organisms. Over the last 25 years, the tutor system created by Memphis University has been utilised by computers to help students with their homework. After typing or speaking an answer, the tutor may determine whether a word or phrase is grammatically or semantically incorrect and explain the reasoning behind the decision. However, badminton education in the United States began a little later than in other countries [4]. According to the researcher, a discussion on the growth trend of badminton in colleges and universities revealed that quality education has become the foundation for talent training; it has become more vital to support the whole development of school's physical and psychological attributes [5]. College students' physical health has been steadily deteriorating over time. With the increasing popularity of badminton, collegiate badminton instruction has become increasingly important [6]. At the same time, as they are working to enhance student learning, educators should work to raise their own technical proficiency and come up with new ways to educate. According to the paper “Study on the Practice of Multi-Level Cooperative Teaching Method,” badminton, particularly doubles, is a sport that involves tacit coordination between two individuals [7]. Because of this, it is recommended that it be implemented in college and university badminton instruction. Innovative teaching approaches and additional teaching practise in fostering students' feelings of unity, collaboration, and enthusiasm for learning are tried. Using the hierarchical cooperative teaching technique, people attempted to use it while instructing college badminton students [8]. Students' attention, initiative, and excitement for learning have increased as a result of the comparison experiments conducted with other classes. These works have offered some references for this article's study, but the experimental findings from prior investigations cannot be reproduced since samples were not used [9].

Existing intelligent educational systems, as well as neural network technology, are briefly summarised and discussed in this article. It depends on instructive objective hypotheses, various keen hypotheses, project reflection hypothesis, mistake back propagation calculation innovation, and the hypothetical model design of the keen-directed badminton framework in view of neural organisation innovation, which establishes the framework for the exploration of badminton [10]. As a result of the development of intelligent learning, there has been an increase in the quantity of semi-structured data in different learning platforms. People arrange and categorise the hidden value underlying different sorts of data. For example, educational data mining is the process of extracting useable information from educational data to better understand students, make educational choices on their behalf, and improve our badminton instruction [11]. An intelligent teaching system is one that uses artificial intelligence and computer technology in a distributed network setting to adaptively organise knowledge resources, execute teaching techniques, offer teaching process services, and perform teaching assessment. In the end, the most important goal of research is to make computer systems smarter [12]. To some degree, computer systems may take the position of human educators in the pursuit of the greatest education, while also reducing the stress on those educators while simultaneously improving educational standards and helping students better grasp the process of self-awareness to enhance the education of students.

Teaching is necessary for the personal growth of pupils, but it is also vital for the well-being of society at large [13]. For this reason, social continuity and development refer to the continuity and development of politics, economics, culture, and technology. People who are capable of developing social politics, economics, culture, and technology must be educated in schools [14]. Teaching is the primary mode of teaching in schools. Schools, in their most basic form, encourage children to get a structured, methodical education. Students may get the information, skills, emotional attitudes and values, etc., and they need to advance social politics, economics, culture, and technology, all of which contribute to social development. This can be accomplished through educational activities. Teaching has a social value that is found on the individual value of teaching and supports the actual existence of society and the future growth of society [15]. Intangible changes to existing educational paradigms have been made as a result of smart education's influence on how pupils learn. The future of education will be dominated by online learning. There are pros and drawbacks to any instructor [16]. A good illustration of this is a teacher who has a lot of experience but is exhausted physically or otherwise. While some instructors are vivacious yet inexperienced, others are quick-witted but unsteady on their feet. There is a greater level of sexism, and some instructors are too cautious and patient. In addition to helping students and instructors identify their own learning and teaching levels, online teaching may also help teachers better leverage their own strengths.

Scientific and reasonable placement of university courses requires extensive study, and this research may be devoted to placing the courses in a scientific and sensible manner. To teach college and university courses, science and sound ideology are essential [17]. Course scheduling in colleges and universities must conform to the “reasonable and practical” principle and include sports education in the current age. Badminton instruction is an essential aspect of a student's education because of current trends in thinking that support the development of students' sports consciousness and self-worth. The most effective learning occurs when students are taught in groups, and this can only be achieved if the group size is appropriate [18]. From our research, people may deduce that the learning outcomes for kids in various groups will vary. Initial recruitment of group leaders and students' self-organisation has been replaced by teacher-guided self-organisation [19]. Grouping is entirely up to you. In a classroom, students who know each other well will form a group. As a result of the group's familiarity with each other, it is difficult to focus on learning since there are too many small talk subjects. To ensure that all students benefit from a positive learning environment, teachers should organise pupils according to their desire to participate [20]. Pretests may help instructors learn about their students' personalities, preferences, abilities, and learning foundations before classifying them. This way, students can benefit from each other's strengths and limitations in their classes. Students with strong organisational abilities and a high level of activity should be chosen as group leaders by instructors since these qualities play an important role in the learning of the other students in the group [21].

Badminton is a kind of major sporting event that attracts large crowds. Almost anybody can play, but there is not a lot of standardisation in the motions. As a result of the limited number of class hours available in college badminton instruction, it is common that no more systematic learning or training takes place once the basics have been introduced [22]. Indirect sparring exercises did not fix any of the pupils' irregular motions, nor did they have the time to lead and methodically finish movement learning. As a result, pupils find classroom learning tedious and monotonous. To finish the teaching process, the instructor must prepare for the final test and examination, and the student will not practise again until after the examination [23]. Due to time constraints, most students are expected to be taught about technology rather than badminton theory as part of the badminton teaching process at colleges and universities.

There are both technical and theoretical sides to badminton instruction, but in college badminton teaching, the technical aspect still holds sway [24]. Venues and equipment are a prerequisite for technical instruction. The majority of collegiate sports fields are still occupied by football, basketball, and volleyball stadiums, despite the fact that the standards for badminton venues and other hardware facilities are far lower than those of other sports [25]. Many badminton sports venues are smaller in size than the three main sports grounds. As a result, many colleges are forced to limit the number of people who participate in badminton activities while maintaining the same number of courses. Additionally, badminton courts and badminton equipment are important roadblocks. It is more practical, direct, and meaningful for pupils to learn badminton [26]. The learning aim is to acquire the fundamentals of badminton and badminton technology, strategies, and basic officiating knowledge. The skill's objective is to be able to grasp fundamental badminton strategies and techniques, as well as physical training, including badminton. To help college students develop healthy lifestyle habits and long-lasting sports concepts, we need to integrate the best aspects of badminton and other team sports like it [27]. Pupils are encouraged to take an active role in their physical well-being and to develop a work ethic and a tenacious character for perseverance in their studies.

### 1.1. Motivation for the Study

The purpose of this study was to provide a mobile sports activity classification system and to perform related learning on sports with in-class interaction and a classification algorithm to accurately recognise sports activities. The mobile system collected sport utilises two portable inertial detecting components accessorised on wireless network-predicated artificial intelligence robots inside the wrist and the ankle of badminton, using a deep decision tree to retrieve its inherent features from the spectrum analyzer of artificial intelligence sport movement transmissions. To gather electrical motion activity created by athletic events, all attendees placed the two portable acceleration sensing modules on their wrist and leg. Following that, we created a complex having to learn sports exercise decision tree (DT) algorithm, which includes sports motion transmission collection, signal preprocessing, sport motion segmentation, transmission normalisation, spectrum analyzer generation, picture merging/resizing, and DT classification to recognise various badminton sports activities.

## 2. Materials and Methods

**Figure 1:**
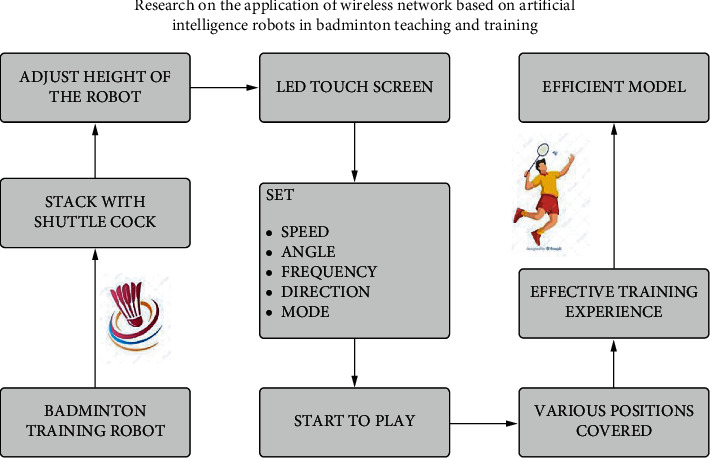
Proposed flow notation for the badminton teaching and learning process.

Badminton is said to be a sport. It is played using rackets to hit the shuttlecock across the net. The main formats of this game are singles and doubles. The movement and aerobic activities of the players play a vital role in the game. The proposed system appoints a badminton training robot to train the players with the skill sets required for the game. It also teaches various techniques that are to be followed for an effective game. The badminton training robot is stacked with shuttlecocks as the initiation process of the training. The badminton court has various areas such as the left service court, right service court, central line, long service line, and back boundary line on each side of the net. The badminton training robot is placed at the centre line on one side with the player on the other side. The robot stands firmly at the fixed spot. The height of the robot is adjusted to match the player. The design with an LED touch screen consists of data that have to be fed by the trainer. The touch screen consists of data such as speed, angle, frequency, direction, and mode. Once the training mode is selected, the robot starts to play. It shoots the shuttlecocks one after another as preprogrammed in the mode. It shoots the shuttlecock in various positions, almost covering the whole court of the opposing player. Wireless sensor networks play a major role in the proposed system. The robot is connected to the network with the help of wireless. The artificially intelligent robot is semi-automatic. It has to be fixed in a position where the court height of the robot has to be adjusted manually. The training modes are set with the help of the LED touch screens by setting the required mode of training. This provides the player with stamina, footwork, aerobic activity, quick response, effective game, etc. Thus, the artificially intelligent robots used in training and teaching badminton to the players resulted in effective training of the players (Figure 1).

The deformation accelerometer and gyroscope measurement methodologies are first calibrated to eliminate responsiveness and offset faults from the raw inertial sensor results. Humans first place the deformation accelerometer on a leveled surface to calibrate it. Following that, the axes of the deformation accelerometer are alternately placed upwards and downwards to correspond with the effect of gravity, which can only be recorded by gyroscope when the moveable inertial identifies its factors as permanent.

This research aids in developing a mobile based learning mechanism for national sports classification scheme and to identify the factors that affects the learning through in-class interactivity, and then a classification algorithm is implemented to accurately identify the individual's sports activities. The mobile platform collected sport uses two handheld inertial detector component magnetic moments on wireless network-based artificial intelligence robots within the wrist and ankle of badminton, with a deep decision tree retrieving unique characteristics from the spectrometer of artificial intelligence athletic movement transmissions. All attendees wore the two transportable acceleration sensor devices on their wrist and leg to collect electrical motion activity generated by sporting events. Then, for anywhere badminton sports activities, designers created an extensive decision tree (DT) algorithm that contains not only sports movement transmission collection, frequency preprocessing, sport movement segmentation, transmission normalisation, spectrum analyzer production, and picture trying to merge but also DT classification.

In particular, wireless network-based artificial intelligence robots for badminton sporting activities with all dimensions of an accelerometer can be gathered and utilised to calibrate raw gyroscope measured data, as shown in equation (1). Sports acuity values within all axes defined in the datasheet could be used to measure navigation system scalars. The average rotation speeds accumulated by initially maintaining a portable inertial significant channel immobile can then be utilised to determine offsets within all rotational axes. At some point, humans will be able to precisely assess the freshest gyroscope measurement data using the component at the time and also offsets as equations.

(1) $$\begin{array}{c} R_{s}=\sum_{n=1}^{K}\left[\begin{array}{ccc} KD_{n} & 0 & 0\\ 0 & KD_{m} & 0\\ 0 & 0 & KD_{p} \end{array}\right]\times \sum_{n=1}^{v}R_{v}+\left[\begin{array}{c} Q_{n}\\ Q_{m}\\ Q_{p} \end{array}\right]. \end{array}$$

where *R*_*s*_ in equation (1) denotes either scaled resonances (*a*_*s*_=[*a*_*sn*_*a*_*sm*_*a*_*sp*_]^*E*^) or angular acceleration  (*ρ*_*s*_=[*ρ*_*sn*_*ρ*_*sm*_*ρ*_*sp*_]^*E*^). *R*_*v*_  represents the fresh amplitude and frequency (*a*_*v*_=[*a*_*vn*_*a*_*vm*_*a*_*vp*_]^*E*^) or rotational motion (*ρ*_*v*_=[*ρ*_*vn*_*ρ*_*vm*_*ρ*_*vp*_]^*E*^). *n* − , *m*−, and *p*–axis of the deformation sensor or gyroscope incorporate the building and are denoted as *KD*_*n*_, *KD*_*m*_and*KD*_*p*_.  *n* − , *m*−, and *p*–axis deviations were represented either by letters *Q*_*n*_, *Q*_*m*_ instead of  *Q*_*p*_  in the deform accelerometer and moreover gyroscope.

A decision tree seems to be a supervised learning technique that can be used to solve both classification and regression problems. However, it is most commonly employed to solve classification issues. It is based on the classification of the surface and quality of the user's game practice with the aid of wireless internet artificial intelligent robots in badminton. These robots makes movement through signals that are received from a variety of users who are inconsistent with the game. During the badminton motion segmentation technique, the vibration interval is split. To reduce the impact of human variation, each badminton motion signal during the sports motion interlude was interpolated into the length of the signal with a more important length. Following that, people are employing strategies to normalise the sport evidence, which proposes that the single amplitude discrepancies between both users are reduced.

The *p* − score standardisation is shown as follows:

(2) $$\begin{array}{c} KR_{x}\left(s\right)=\sum_{n}^{m}\frac{\prod KR\left(s\right)-\prod KR_{\text{mean}}}{\prod KR_{\text{std}}}. \end{array}$$

where *KR*(*s*) and *KR*_*x*_(*s*) are indeed the original and normalised sport motions, respectively, that really are signals within the badminton motion period. The amount of times steps inside a sport motion interval is denoted by  *s*. The variation of the sports motion signals inside the badminton movement timeframe is represented by *KR*_mean_and*KR*_std_, correspondingly.

When all of the information from the spectrogram was available, a transformation function is performed to spread it along the temporal message into small equal sample lengths, but then the discrete wavelet transform is quantified separately for each group. The DT of the normalised badminton motion signal is explicitly defined with the following equation:

(3) $$\begin{array}{c} DT\left\{n\left[x\right]\right\}\left(y,\rho \right)=\\ N\left(y,\rho \right)=\sum_{x=-\varphi }^{\varphi }n\left[x\right]\rho \left[x-y\right]e^{-j\rho x}. \end{array}$$

The decision tree is represented by *DT*, where *n*[*x*] denotes the normalized input for the badminton training, [*φ*] denotes the pixel value, and  *y* is the window executable centre. *DT* is calculated with an 8128-point *ρ* transform and a modulated signal that overlaps with 60% accuracy. The retrieved *N* features are defined as the amplitude that divides the square of the *DT* from normalised badminton signals, as shown in the following equation:

(4) $$\begin{array}{c} \sum_{n=1}^{x=1}\text{spectrom}\left\{n\left[x\right]\right\}\left(y,\rho \right)=\sum_{y=1}^{N}{\left|N\left(y,\rho \right)\right|}^{2}. \end{array}$$

The properties produced by convolutional *m* are utilised to establish its output data of the convolution operation and have the same picture resolution as the convolutional layers' data input. The output signals of the convolutions layers are referred to as features extracted and thus are depicted as follows:

(5) $$\begin{array}{c} s_{ij}^{I,s}=\int \left(h_{s}^{I}+\sum_{y=1}^{X}U_{y,x}^{I,s}n_{i+y-1j+x-1}^{I-1,s}\right). \end{array}$$

From equation (5), *l* relates to a surface indicator, *U* and *n* are the shape and size of the executable environment size, *h*_*s*_^*I*^  refers here to decision making for such *s*^*th*^ subspace of this *I*^*th*^ layer, *U*_*y*,*x*_^*I*,*s*^  is the density between both the information *n*_*i*+*y*−1*j*+*x*−1_^*I*−1,*s*^ and the surface living quarters of an Ith layer, and ∫(*n*) itself is given in the following equation:

(6) $$\begin{array}{c} \int \left(n\right)=\sum_{n=1}^{n}\text{max}\left(0,n\right). \end{array}$$

The equation is the above derived form of  *G*_*ij*_^*I*,*s*^. The convolution technique employed in this publication is max(0, *n*)  max-pooling, which also produces the maximum value from adjacent feature extraction and can be determined using the following equation:

(7) $$\begin{array}{c} G_{ij}^{I,s}=\underset{o\in O}{\max}\left(s_{i\times M+o,j\times M+o}^{I,s}\right). \end{array}$$

For which *O*=2 × 2 represents the accumulation size and *M*=2 represents the redistribution running style. The information for such entire layer with the underlying features are represented as *Q*_*b*_^*I*^, and *u*_*bP*_ is also recovered from the densely compacted completely linked layers. *u*_*bP*_^*I*−1^ convolution layer has been destroyed, resulting in the one-dimensional decision tree classification algorithm illustrated in the following equation:

(8) $$\begin{array}{c} Q_{b}^{I}=\int \sum_{P}\left(u_{bP}^{I-1}\right)+h_{b}^{I}. \end{array}$$

The third crucial frames performance, softmax, assesses a prior probability and forecasts sport various classes.

(9) $$\begin{array}{c} G\left(s|Q\right)=\underset{s\in S}{\text{argmax}}\frac{\text{exp}\left(Q_{b}^{d}\right)}{\sum_{y=1}^{X_{S}}\text{exp}\left(Q_{y}^{d}\right)}. \end{array}$$

The DT algorithm is being used to keep updating the system parameters in order to reduce a classification pass optimisation algorithms in equation (9), where *s* ∈ *S* represents the sport fitness class, *X*_*S*_ represents the total number of sport classroom instruction, and *d* represents the very last surface indicator during the training phase.

To evaluate the efficacy of the suggested precision learned in school badminton activity DT-based algorithm, the following equation is used:

(10) $$\begin{array}{c} \text{accuracy}\left(\%\right)=\frac{\text{TP}+\text{TN}}{\text{TP}+\text{TN}+\text{FP}+\text{FN}}, \end{array}$$

where TP stands for true positive, TN stands for inadequate performance, and FP stands for false positive, while FN stands for false negative. Following the below equation, the accuracy in the learning classroom is classified.

(11) $$\begin{array}{c} K_{g}\left(\%\right)=\frac{\text{TP}}{\text{TP}+\text{FP}}\times 100. \end{array}$$

In the following equation, *K* is the set of the classes that is utilised to analyze the overall classification performance.

(12) $$\begin{array}{c} K_{e}\left(\%\right)=\frac{\text{TP}}{\text{TP}+\text{FN}}\times 100. \end{array}$$

Furthermore, as shown in the following equation, the evaluation of classification accuracy of the classroom education for the activities is represented.

(13) $$\begin{array}{c} CV\left(\%\right)=\frac{\sum_{i=1}^{S}{\text{TP}}_{i}}{\text{TP}+\text{TN}+\text{FP}+\text{FN}}\times 100. \end{array}$$

The DT classification algorithm uses CV cross-validation methods to correctly categorise badminton actions as follows:

(14) $$\begin{array}{c} K_{e}\left(\%\right)=\sum_{y=1}^{N}{\left|N\left(y,\rho \right)\right|}^{2}+\sum_{i=1}^{S}TP_{i}, \end{array}$$

(15) $$\begin{array}{c} CV\left(\%\right)=\;\sum_{T⟶F}^{N}\text{TP}+\text{TN}+\text{FP}+\text{FN}+\sum_{y=1}^{N}{\left|N\left(y,\rho \right)\right|}^{2}. \end{array}$$

It is a transportable classification system, and it is engaged in dealing with specific application learning as follows:

(16) $$\begin{array}{c} K_{e}\left(\%\right)=\sum_{T⟶F}^{N}\frac{\text{TP}}{\text{TP}+\text{FN}}\times 100+\sum_{y=1}^{N}{\left|N\left(y,\rho \right)\right|}^{2}. \end{array}$$

The maximum rotation speeds obtained by initially keeping a transportable inertial significant channel motionless can then be used to calculate offsets in all rotational axes. Humans will eventually be able to precisely judge the most recent gyroscope measured data using the components at a time and offsets as equations.

## 3. Result and Discussion

Decision tree appears to be a supervised learning strategy for solving classification and regression problems. It is, nevertheless, most typically used to tackle categorisation problems. Based on that, a variety of individuals who are inconsistently monitoring the game send signals to classify the surface and quantity of wireless Internet artificial intelligence robots in badminton. The vibration interval is separated, the same as in badminton motion segmentation. Each badminton motion signal during the sports motion interlude was interpolated into the duration of the signal with more important length to lessen the impact of human variation. Following that, people are using ways to normalise the sport evidence, implying that the single amplitude differences between both users have decreased.

**Figure 2:**
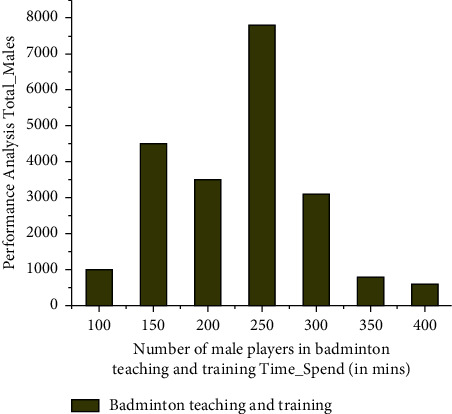
Time spent by number of male players in badminton teaching and training.

The number of male players engaging in exercising training for participation in wireless network-based artificial intelligence robots for badminton is depicted. *R*_*s*_ denotes either scaled resonances (*a*_*s*_=[*a*_*sn*_*a*_*sm*_*a*_*sp*_]^*E*^) or angular acceleration (*ρ*_*s*_=[*ρ*_*sn*_*ρ*_*sm*_*ρ*_*sp*_]^*E*^). *R*_*v*_  represents the fresh amplitude and frequency (*a*_*v*_=[*a*_*vn*_*a*_*vm*_*a*_*vp*_]^*E*^) or rotational motion (*ρ*_*v*_=[*ρ*_*vn*_*ρ*_*vm*_*ρ*_*vp*_]^*E*^). *n* − , *m*−, and *p*–axis of the deformation sensor or gyroscope incorporate the building and are denoted as *KD*_*n*_, *KD*_*m*_and*KD*_*p*_ based on this to retrieve in Figure 2.

This badminton training necessitates the players' undivided attention and effort, and thus, the training should meet those requirements. Furthermore, as the amount of players grows, schedule must be set, and thus, all players will benefit through training. The players were capable of engaging in training for a maximum of five hours, as seen in the accompanying figure. In addition, for four hours, the maximum percentage of male players was available. A total of approximately 8,000 players were instructed over the course of four hours. Hundreds of players, though, were able to train for further than five hours. Because this study does not really focus on the team's resting length, it is assumed that the player is already in continuous training. It is also assumed that its data were gathered with the use of fitness apps and wearables that operate on the artificial intelligence idea.

**Figure 3:**
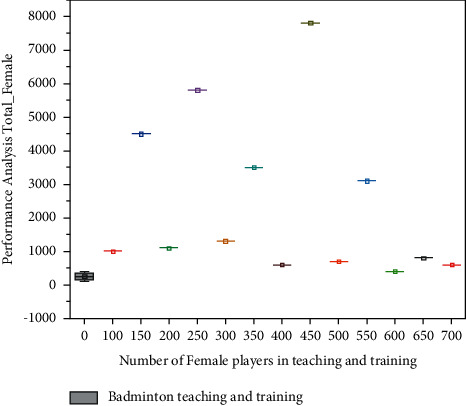
Time spent by number of female players in teaching and training.

Female gamers, like male players, have a difficult time getting trained. Training can take place at the coach's location or even in a remote area using AI equipment and wireless networking. Throughout this training mechanism, both players and coach use the most up-to-date information technological concepts, such as AI, with the assistance of mobile applications for interactive sessions or live monitoring of the players' activity. The accompanying graph demonstrates that the greatest number of women players has managed to train for further than six hours, while the overall quality is less than five hours. The athletes in both incidents of male and female badminton training were thought to be engaged in two distinct gyms in China. The overall number of male and female players both from gyms is evaluated for study when *KR*(*s*) and *KR*_*x*_(*s*)  are indeed the original and normalised sport motions, respectively, that really are signals within the badminton motion period. The amount of times steps inside a sport motion interval is denoted by  *s*. The variation of the sports motion signals inside the badminton movement timeframe is represented by *KR*_mean_  and  *KR*_*std*_, respectively, and corresponding results are given in Figures 2 and 3.

**Figure 4:**
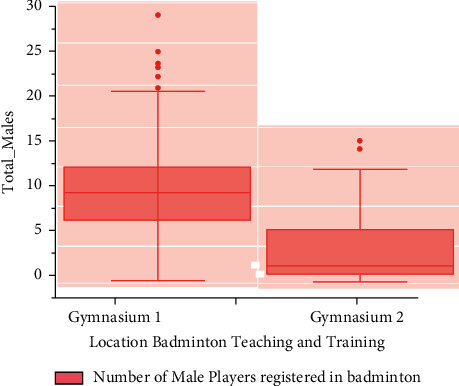
Number of male players registered in badminton teaching and training gym.

**Figure 5:**
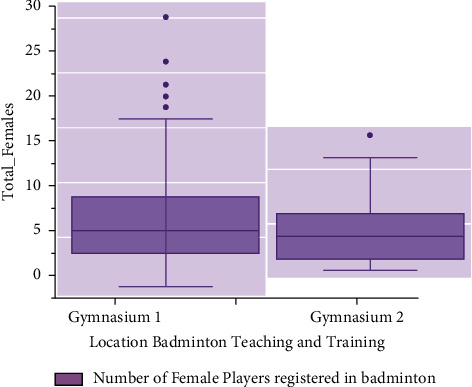
Number of female players registered in badminton teaching and training gym.

To a surface indicator, *U*and*n* are the shape and size of the executable environment size, *h*_*s*_^*I*^  refers here to judgemental for such *s*^*th*^ subspace of *I*^*th*^ layer, *U*_*y*,*x*_^*I*,*s*^  is the density between both the information *n*_*i*+*y*−1*j*+*x*−1_^*I*−1,*s*^ and the surface living quarters of an *I*th layer, and ∫(*n*) itself is based on this to retrieve in Figures 4 and 5 that show the number of male and female players who have registered for training in the first gym. The reason for going to first gym could be the accessibility of the greatest instructor, proximity to their living place, flexible training times, and so on. This does not imply that now the second gym is less important. The number of players registered to badminton training is now in the thousands.

**Figure 6:**
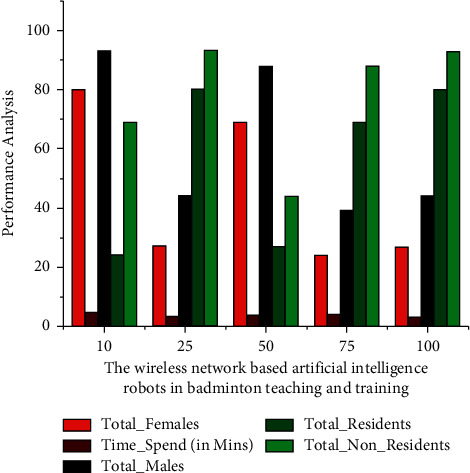
Confusion matrix for the wireless network-based artificial intelligence robots in badminton teaching and training.

The number of participants involved in badminton training was anticipated in five categories: female, male, Chinese residents, nonresidents of China, and the overall number of players involved in training. A matrix was used to calculate the suggested DT recognition accuracy. They had used statistics to show 5 various badminton visuals, including having served, groundstrokes rubbing, and backhand. The discriminant function of the experimental data reported in this research is depicted for which *O*=2 × 2 represents the accumulation size and *M*=2 represents the redistribution running style. The information for such entire layer is represented as *Q*_*b*_^*I*^, and *u*_*bP*_ is also recovered from the densely compacted completely linked layers. *u*_*bP*_^*I*−1^ convolution layer has been destroyed, resulting in the one-dimensional decision tree classification algorithm illustrated in Figure 6. In five different ball-hitting movement categorisation approaches, its decision tree algorithm achieved an accurate recognition accuracy of 95%, indicating its usefulness.

The number of students involved in various activities is increasing day by day, among which, badminton has increased in popularity benefits in the form of less regulations on the field and the ease with which it can be learned (refer Table 1). This study developed a health monitoring sports activity classification method that can accurately recognise badminton behaviour. A separate specific type affixed to a portion of a badminton racket implement is used to collect data on badminton activities. The extracting feature decision tree classification technique is used to calculate its goal—scoring signal. It compares the existing process for having trained badminton sporting activities in men (69%) and women (24%), based on time for men (0.78%) and women (0.39%) in terms of overall exactness of men (83%) and women (29%). In our decision tree approach, we get the leading training badminton sporting activities in men (80%) and women (27%) based on time for men (0.88%) and women (0.27%) based on the overall accuracy of men (93%) and women (44%). The number of pupils participating in various activities is growing every day. Badminton, for example, has grown in popularity as a result of less constraints on the court and the ease with which it can be learned (refer Table 1). This research created a health monitoring sports activity classification algorithm that accurately recognises badminton play. Data on badminton activities are collected using a separate particular type affixed to a piece of a badminton racket implement. The extracted features are incorporated in the goal - scoring signal - and is calculated using a decision tree classification algorithm.

## 4. Conclusions

Artificial intelligence has already made its mark on a number of businesses. In the same way, it has been utilised in the sports industry to train athletes by teaching them the skill set essential for the sport. Badminton education and training could benefit from the use of wireless networks based on artificial intelligence robots. People have proposed a system in which athletes would be taught badminton by intelligent robots. Multiple playsets were preloaded onto the robot. The trainer has to choose essential elements such as the training mode and the set height required for a certain player in the robot. This robot will use a variety of training methods to help the players improve with the changes in variables such as speed, frequency, angle, height, and so forth. Robotic training and instruction have been found to be more effective than human training. This research used a decision tree algorithm, which helps students learn how to make consistent choices through this method. The proposed model has provided an accuracy of 93%. In future research, it is highly recommended to evaluate the efficiency of implementing deep learning model in teaching and training of badminton.

## Figures and Tables

**Table 1:** Comparison analysis for the intelligence in badminton sports activities.

Algorithm	Training badminton sports activities (%)		Time (s)	Speed (%)	Accuracy (%)
Decision tree classification algorithm	Male	80	0.86	90	93
	Female	27	0.27	90	44

Existing method: sports motion segmentation method	Male	69	0.78	83	88
	Female	24	0.29	83	39

## Data Availability

The data used to support the ﬁndings of this study are available from the corresponding author upon request.
